# Sociodemographic determinants and assessment of anti-α-Gal IgG titers in head and neck cancer patients

**DOI:** 10.3389/fonc.2025.1686122

**Published:** 2025-12-03

**Authors:** Dayse Marcielle de Souza Lopes, Clarissa Fernandes Gomes, Cristina Paixão Durães, Larissa Lopes Fonseca, Sarah Patrícia Souza Costa, André Luiz Sena Guimarães, Sônia Ribeiro Arrudas, Guilherme Araújo Lacerda, M. G. Finn, Sílvio Fernando Guimarães de Carvalho, Ana Paula Venuto Moura, Alexandre Ferreira Marques

**Affiliations:** 1 Universidade Estadual de Montes Claros – Unimontes, Programa de Pós-graduação em Ciências da Saúde (PPGCS), Montes Claros, MG, Brazil; 2 Universidade Estadual de Montes Claros – Unimontes, Programa de Pós-graduação em Biotecnologia (PPGB), Montes Claros, MG, Brazil; 3 Universidade Estadual de Montes Claros – Unimontes, Departamento de Biologia Geral, Montes Claros, MG, Brazil; 4 Universidade Estadual de Montes Claros – Unimontes, Departamento de Odontologia, Montes Claros, MG, Brazil; 5 School of Chemistry and Biochemistry, Georgia Institute of Technology, Atlanta, GA, United States; 6 Universidade Estadual de Montes Claros – Unimontes, Departamento de Saúde da Mulher e da Criança, Montes Claros, MG, Brazil; 7 Center for Molecular and Cellular Biosciences, School of Biological, Environmental, and Earth Sciences, University of Southern Mississippi, Hattiesburg, MS, United States

**Keywords:** alpha-gal, head and neck neoplasms, virus-like particles, carbohydrates, cancer

## Abstract

Head and neck cancer (HNC) remains a pressing global health challenge, particularly in regions with lower socioeconomic status, where risk behaviors such as tobacco use and alcohol consumption are prevalent. Despite advances in treatment, reliable biomarkers for the early detection and monitoring of HNC remain lacking. The α-Gal carbohydrate epitope, absent in humans but present in other mammals, has garnered interest due to the natural presence of anti-α-Gal antibodies in the human immune repertoire, comprising approximately 1% of total circulating IgG. We investigated the role of anti-α-Gal IgG as a potential biomarker by performing ELISA on serum samples from a cohort of 11 patients diagnosed with squamous cell carcinoma (SCC) of the head and neck. Eight were older male patients, most of whom lived in rural areas and engaged in manual occupations. High rates of tobacco (81.8%) and alcohol consumption (63.6%) were observed, in line with established risk factors for HNC. These individuals showed significantly elevated anti-α-Gal antibody titers compared to non-cancer controls. Chemotherapy with cisplatin did not markedly affect antibody levels, suggesting consistent immune reactivity across treatment status. These results suggest that anti-α-Gal antibodies may serve as promising biomarker candidates in HNC and warrant further investigation to clarify their potential diagnostic and immunotherapeutic applications.

## Introduction

Head and neck cancer (HNC) is a prevalent global issue in terms of incidence and mortality. According to the National Cancer Institute (NCI), it ranks second in incidence among men (1, 2). The incidence of HNC is higher in regions with low socioeconomic development, attributed to prevalent behavioral and lifestyle patterns in these areas, which contribute to prolonged exposure to risk factors, leading to late diagnosis, difficulty accessing therapeutic resources, and high lethality rates (2–4). HNC patients often exhibit unique biomarkers that can assist in diagnosis and prognosis, including carbohydrate antigens (5–7). Glycoproteins such as galectins and mucins (e.g., MUC1) have been investigated for their roles in tumor progression and metastasis, given their involvement in cell-cell and cell-matrix interactions (8, 9). Additionally, carbohydrate antigens like CA19–9 and sialylated glycoproteins have shown potential for disease monitoring (10–12). These proteins and carbohydrate-based markers are essential in understanding tumor biology and may serve as targets for novel therapeutic interventions, though they still require extensive research and clinical validation.

The α-Gal epitope (galactose-α-1,3-galactose) is a carbohydrate structure abundantly expressed on glycoproteins and glycolipids of non-primate mammals (13). In humans, the gene encoding the enzyme α1,3-galactosyltransferase, responsible for synthesizing the α-Gal epitope, is inactive. Consequently, humans naturally produce anti-α-Gal antibodies, constituting approximately 1% of circulating immunoglobulins (14, 15). Although the α-Gal epitope has not been prominently associated with MUC1, similar carbohydrate antigens, such as the GalNAc structures found in the Tn and sialyl-Tn antigens, are frequently observed in cancer-associated MUC1^12^. These altered glycosylations are critical in tumor immunogenicity and can influence interactions with the immune system, similar to how α-Gal can impact immune responses in other contexts.

In anticancer immunology, the interaction between the α-Gal epitope and anti-α-Gal antibodies has been harnessed to enhance tumor immunogenicity. By modifying autologous tumor cells or membranes to express α-Gal epitopes, these cells can be targeted by anti-α-Gal antibodies (16). This targeting facilitates the binding of the Fc region of the antibodies to Fcγ receptors on antigen-presenting cells (APCs), promoting effective uptake and processing of tumor-associated antigens. Subsequent presentation of these antigens leads to the activation of tumor-specific T cells, potentially eliciting a robust anti-tumor immune response (15, 17). Furthermore, the α-Gal epitope has been utilized in developing synthetic glycolipids, such as AGI-134, which, upon intratumoral injection, convert tumors into *in situ* autologous vaccines (18). This approach induces systemic anti-tumor immunity by promoting the destruction of tumor cells and facilitating their uptake by APCs, thereby enhancing the presentation of tumor antigens and stimulating a systemic immune response against residual tumor cells (17). Despite advances in harnessing the α-Gal epitope to enhance tumor immunogenicity, the role of α-Gal and anti-α-Gal antibodies in head and neck cancer (HNC) remains unexplored. HNC is a malignancy characterized by profound immune evasion mechanisms and a high incidence of late-stage diagnosis, underscoring the urgent need for novel biomarkers and therapeutic targets. Aberrant glycosylation patterns, including the expression of tumor-associated glycans, are well-recognized hallmarks of cancer progression and immune modulation, as highlighted in foundational reviews on cancer-associated glycans and their immunogenicity (19, 20). However, the potential contribution of α-Gal to this landscape has not been systematically evaluated in HNC.

Given the immunogenic nature of the α-Gal epitope and its established role in modulating immune responses, we hypothesized that anti-α-Gal IgG antibodies are elevated in HNC patients, potentially reflecting an immune reaction to tumor-associated carbohydrate antigens. To investigate this, we identified a small but well-characterized cohort of HNC patients in Minas Gerais, Brazil, and described their sociodemographic and clinical characteristics. We then evaluated anti-α-Gal IgG levels. While the limited cohort size precludes broad generalizations, the data reveal promising potential for anti-α-Gal both as a diagnostic marker and as a target for immunotherapeutic strategies, thereby justifying larger studies.

## Method

### Ethical aspects

This study was approved (opinion number 3.379.334/2019) by the Research Ethics Committee of the State University of Montes Claros - CEP/Unimontes, accredited by CONEP - National Research Ethics Committee. Ethical precepts were adopted in accordance with Resolution 466/12 of the National Health Council.

### Study location and population

The investigation was conducted in Montes Claros, a municipality situated in the Upper Middle São Francisco Basin, southeastern Brazil, and in the northern part of Minas Gerais state. The population of Montes Claros is estimated to be 414,240 inhabitants, and the municipality is situated at a latitude of 16° 43’ 41”, longitude 43° 51’ 54”, and an altitude of 638 meters.

This quantitative study was conducted with 11 individuals (of all genders, aged 18 or older) diagnosed with HNC, as determined by clinical and laboratory examinations. The patients were deliberately chosen after visiting the Dilson Godinho Hospital in Montes Claros for medical consultation, fulfilling the study’s inclusion and exclusion criteria, and signing the Informed Consent Form (ICF). The participants were assessed in two phases: before and during the treatment protocol suggested by the Ministry of Health, which consists of chemotherapy with cisplatin combined with radiotherapy between July and September 2019. Patients with a positive serological test for acquired immunodeficiency syndrome (HIV), expectant mothers, and those who were extremely weak were excluded from the research. Three healthy individuals (negative for HNC and Chagas disease) were included in the analysis to provide control samples.

### Collection of sociodemographic, epidemiological, and clinical data

The following variables were collected: sex, age, origin, education, occupation, exposure to the sun, consumption of hot beverages, smoking, alcoholism, and family history of cancer. In addition, the patient’s diagnosis and treatment protocol to which he was submitted were evaluated, taking into account the inclusion or exclusion of chemotherapy treatment. All information was obtained from medical records, anamnesis forms, and questionnaires.

### Biological samples

Eleven patients were selected for this study, comprising six individuals with HNC who had received chemotherapy and five individuals with HNC who had not undergone chemotherapy. The qualified professional who conducted the study collected 4 mL of whole blood from each participant using the venipuncture technique. To ensure sterility, the researcher utilized sterile needles and syringes, adult tourniquets, collection tubes with clot activator, and cotton for phlebotomy. After collection, the samples were centrifuged at 4000 RPM to separate the serum. The researcher collected the serum, approximately 2 mL, using a single-channel micropipette and discarded the clot appropriately. The serum samples were then stored at a temperature of −20°C.

### Serological evaluation – ELISA

The serological evaluation using ELISA was carried out at the macro-regional laboratory of Montes Claros – Regional Health Superintendence. The serum of patients with HNC was used for measuring IgG anti-α-Gal antibodies, and the Qβ (α-Gal)_540_ particle served as the antigen in the plaque. This particle has been previously described for its diagnostic and immunological functions in the context of leishmaniasis and *T. cruzi* infection (21, 22). Its polyvalent nature effectively presents the small-molecule α-Gal epitope for antibody recognition. For positive and negative controls, a pool of sera from Chagasic patients (5 samples) who had high titers of IgG antibodies and anti-α-Gal was used, as well as a pool (3 samples) from individuals who were negative for Chagas disease and did not have HNC.

96-well polystyrene ELISA plates from Sarstedt^®^ were coated with Qβ(α-Gal)_540_ antigens at a concentration of 10 ng/mL in phosphate-buffered saline (PBS), pH 7.6, for 16–18 hours at 4°C. Following the overnight incubation, the free sites of epitope binding were blocked with a 2% lecithin solution in phosphate-buffered saline (PBS) for 50 min at 37°C. After blocking, the plates were washed to remove excess solution. The antigen-coated plates were then sequentially incubated with sera in duplicate at 1:100, 1:200, 1:400, and 1:800 dilutions in PBS containing 0.2% lecithin for 90 min at 37°C. Following this, biotinylated anti-human IgG secondary antibody was added at a dilution of 1:4000 in PBS, and the mixture was incubated for 30 min at 37°C. Subsequently, streptavidin was added at a dilution of 1:3000 in PBS and incubated for 30 min at 37°C. After each incubation step, the plates were washed three times with PBS/0.05% Tween solution and then dried by inversion on absorbent paper. OPD (orthophenylenediamine) substrate was added, and the reaction was allowed to develop for 15–20 min at room temperature in the dark before treating with 2N sulfuric acid to stop the reaction. Absorbance was measured at 490 nm.

### Statistical analyses

Statistical analyses were performed in GraphPad Prism v9.5.1 (GraphPad Software, La Jolla, CA). Two-tailed α=0.05 was used throughout. For each serum dilution, data were first assessed for normality (Shapiro–Wilk) and homogeneity of variances (Brown–Forsythe). When assumptions were met, we used one-way ANOVA to compare the three groups (NCD, CDU, CDT), followed by multiplicity-adjusted *post hoc* tests. Planned pairwise contrasts (CDU vs NCD; CDT vs NCD) were evaluated with Welch’s t-tests (unequal variances allowed) and adjusted for multiple testing using the Holm–Bonferroni procedure (conservative control of family-wise error). If ANOVA assumptions were not met or group sizes were <5, we performed a sensitivity analysis using Kruskal–Wallis with Dunn’s *post hoc* tests (Bonferroni-adjusted). Exact P values, standardized effect sizes (Hedges’ g for pairwise contrasts), and 95% CIs are reported where applicable. Error bars in figures represent SD (not SEM) for consistency. Pooled control note: When the NCD control consisted of a pooled sample (single composite value), inferential comparisons were not performed due to the absence of within-group variance; such points are presented descriptively only. Interpretation caveat: Given the limited sample size (n=11 patients; small control set), all statistical outputs are interpreted as exploratory signals rather than definitive differences and will require confirmation in larger, matched cohorts.

## Results

### Socioeconomic and cultural factors

Among the 11 patients diagnosed with head and neck cancer (HNC) included in this study, 8 (72.7%) were male, with an age range of 45 to 81 years and a mean age of 56 years. Most participants (72.7%) were under 60 years of age. Regarding educational attainment, nine patients (81.8%) had attended only elementary school, with some not completing it fully. Rural occupations were predominant, with four (36.4%) working as farmers and two (18.2%) as bricklayers (**Table 1**).

**Table 1:** Sociodemographic characteristics of the study population.

Sociodemographic characteristics	Category	Absolute frequency (n)	Relative frequency (%)
Sex	Male	8	72.7
	Female	3	27.2
Age group	Adult 45 - 59	8	72.7
	Elderly 62 - 81	3	27.2
Education	Elementary school	9	81.8
	High school	2	18.1
Occupation	Farmer	4	36.3
	Bricklayer	2	18.1
	Household	1	9.0
	Policeman	1	9.0
	Personal trainer	1	9.0
	Truck driver	1	9.0
	Pensioner	1	9.0

### Risk factors

Regarding tobacco and alcohol consumption, nine (82.8%) of the patients were former smokers, and 8 (72.7%) were former drinkers, although the quantities consumed were not assessed (**Table 2**). Notably, 8 (72.7%) of the patients reported habitual sun exposure. A family history of cancer appeared to have limited influence, being reported by only two (18.2%) of the participants. Additionally, consumption of hot beverages was reported by two individuals, while another two denied such consumption, and seven responses (63.6%) were undetermined.

**Table 2:** Risk factors associated with cancer.

Risk factors	Category	Absolute frequency (n)	Relative frequency (%)
Smoking	Former smoker	9	81.8
	Never smoked	2	18.1
Alcoholism	Former drinker	8	72.7
	Never drank	3	27.2
Exposure to the sun	Positive	8	72.7
	Undetermined	3	27.2
Family history	Positive	2	18.1
	Negative	5	45.4
	Undetermined	4	36.3
Hot drink consumption (Coffee)	Positive	2	18.1
	Negative	2	18.1
	Undetermined*	7	63.6

Undetermined*: The patient did not respond clearly.

### Clinical characterization

The findings presented in **Table 3** reveal a varied distribution of head and neck cancer (HNC) sites, with the highest incidence observed in the oral floor, oropharynx, and base of the tongue, each accounting for 18.1% of cases. As expected, the predominant histological type identified was squamous cell carcinoma, aligning with established patterns for this type of malignancy.

**Table 3:** Clinical characteristics of the 11 patients analyzed in this study.

Patient	Anatomical site
1	Oropharynx
2	Larynx
3	Tongue base
4	Nasal sinus
5	Retromolar region
6	Oropharynx
7	Lower left gum
8	Buccal floor
9	Tongue base
10	Floor of mouth
11	Soft palate

### Evaluation of the production of anti-α-Gal IgG antibodies

**Figure 1:**
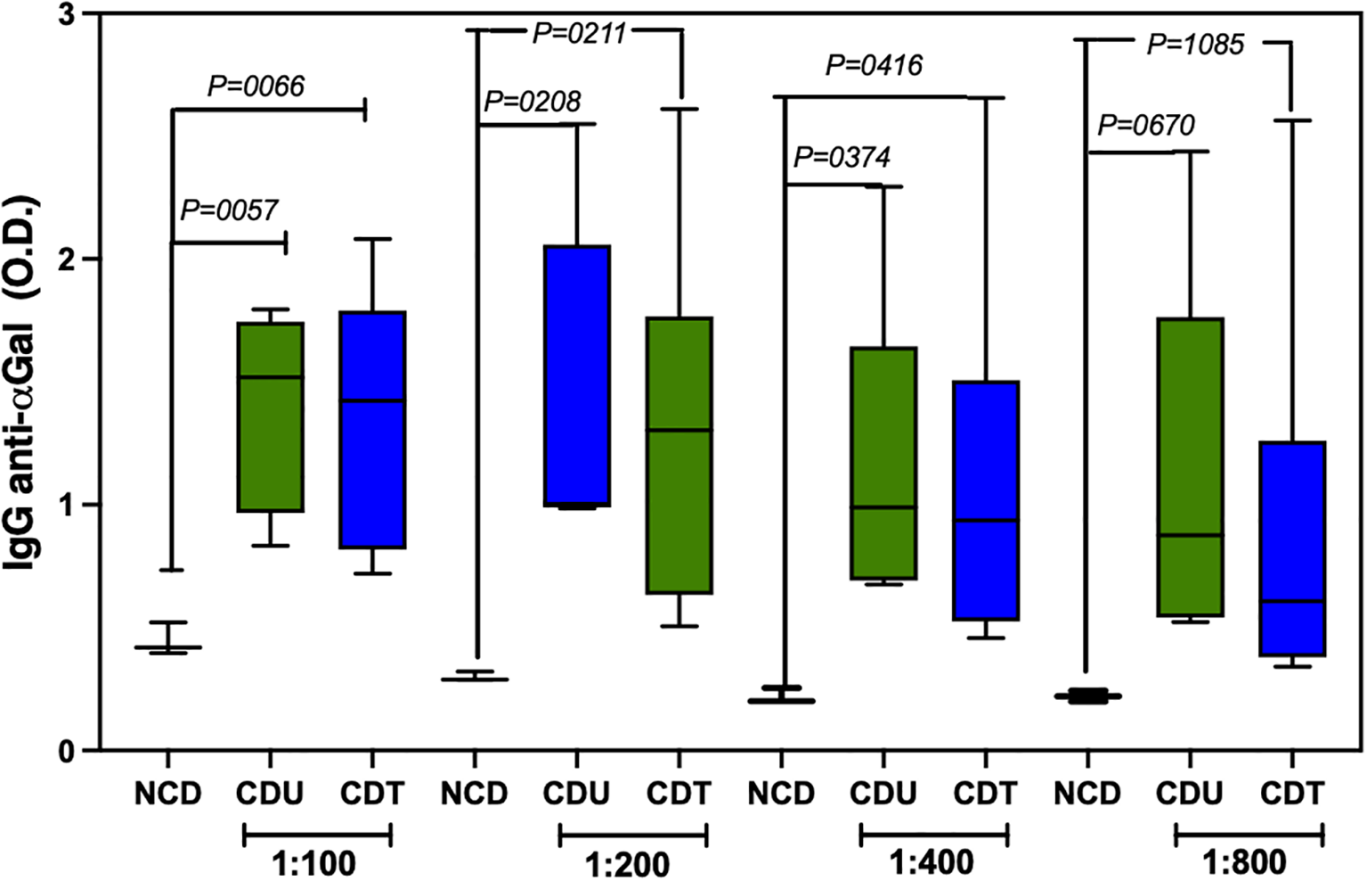
Anti-α-Gal IgG antibody titers in human serum samples across clinical groups. ELISA was performed to assess anti-α-Gal IgG levels at serial serum dilutions (1:100, 1:200, 1:400, and 1:800) in three cohorts: non–cancer diagnosed (NCD; pooled samples from three individuals), cancer diagnosed untreated (CDU), and cancer diagnosed treated (CDT). Bars represent mean optical density (OD) values ± SEM at each dilution. Statistical comparisons were performed using ANOVA with Bonferroni correction; P values indicate differences between NCD and each cancer group (CDU, CDT).

Anti-α-Gal IgG titers were significantly elevated in both cancer-diagnosed groups, untreated (CDU) and treated (CDT), compared to the non-cancer diagnosed (NCD) group, across all tested serum dilutions (1:100 to 1:800). The highest antibody levels were observed in the CDU group, indicating a heightened immune response to α-Gal epitopes in the presence of active disease. Notably, anti-α-Gal titers in the CDT group remained elevated and were not substantially reduced following chemotherapy, suggesting that anti-α-Gal responses persist despite treatment (**Figure 1**). These findings support the potential of anti-α-Gal IgG as a potential immunological biomarker in head and neck cancer and warrant further investigation into its role in tumor-associated immune activity and cancer immunosurveillance.

## Discussion

Head and neck cancer (HNC) continues to represent a critical global health challenge, particularly in regions with lower socioeconomic development. Its etiology is multifactorial, influenced by high-risk behaviors, environmental exposures, and socioeconomic factors (23, 24). Tobacco and alcohol consumption remain the primary modifiable risk factors for HNC, with strong evidence linking these habits to cancers of the larynx and oral cavity (25). In this study, 72.7% of participants reported tobacco and alcohol use, aligning with existing literature and emphasizing the ongoing need for public health interventions targeting these behaviors.

The sociodemographic findings from this study align with prior research, showing a predominance of HNC cases in males (72.72%) and older adults, with a mean age of 56 years. These observations are consistent with the findings that highlight age and male gender as critical risk factors for HNC (26, 27). While the gender disparity remains significant, rising tobacco and alcohol consumption among women suggests a narrowing gap in HNC incidence between genders (28, 29). Additionally, the study revealed a strong association between rural occupations and HNC, with 36.36% of patients being farmers. Prolonged sun exposure and occupational contact with carcinogens likely exacerbate cancer risks in these populations. These findings underline the importance of tailoring interventions to address the specific environmental and occupational risks faced by rural communities. Interestingly, family history contributed minimally to HNC risk in this study, with only 18.1% of cases reporting a familial link. This reinforces the notion that individual behaviors and environmental exposures are more significant determinants of HNC risk than genetic predisposition. This supports broader epidemiological trends indicating that while genetic factors play a role in cancer etiology, lifestyle and environmental factors often outweigh hereditary influences in HNC development (30, 31).

The elevated levels of anti-α-Gal IgG antibodies observed in patients with head and neck cancer (HNC), particularly those with squamous cell carcinoma (SCC), may reflect a natural component of the host’s immune surveillance mechanism. Unlike tumor-promoting antibodies, anti-α-Gal antibodies are naturally present in humans due to continuous exposure to gut microbiota and environmental antigens expressing the α-Gal epitope (32), which is absent in human tissues but ubiquitous in non-primate mammals and certain microbes (33). The increased anti-α-Gal response in untreated cancer patients may signify an ongoing immune recognition of tumor-associated glycans or microbial mimics rather than a pathological or harmful process. Notably, the persistence of elevated anti-α-Gal antibody levels in chemotherapy-treated patients suggests that standard treatment regimens do not significantly diminish this immune response. However, due to the limited sample size, it remains unclear whether treatment modulates anti-α-Gal responses in a meaningful way or if antibody levels fluctuate in relation to tumor burden, immune status, or systemic inflammation. Further longitudinal studies are needed to disentangle these factors.

Far from being detrimental, the anti-α-Gal response may represent an immunological advantage. Preclinical studies have shown that α-Gal-based vaccines and glycolipids can enhance anti-tumor immunity by promoting the activation of dendritic cells and T cell responses (34, 35). Moreover, the presence of anti-α-Gal antibodies has been linked to improved outcomes in some cancers, including colorectal carcinoma, where their binding to α-Gal-decorated tumor cells facilitated immune-mediated clearance (35). These findings support the notion that anti-α-Gal antibodies could serve as a valuable component of tumor immunosurveillance and a potential adjunct in cancer immunotherapy.

In the context of HNC, a disease often marked by immune evasion and late-stage diagnosis, integrating immune-based markers such as anti-α-Gal IgG with conventional glycoprotein biomarkers could enhance diagnostic accuracy and patient stratification. Although the present study relied on a small and not optimally matched control group, we have emphasized this limitation and consider these findings exploratory. Future studies incorporating larger, demographically and clinically matched cohorts will be critical to validate the specificity of anti-α-Gal responses in HNC. Taken together, our findings suggest that anti-α-Gal IgG holds promise not only as a diagnostic and prognostic biomarker but also as a potential immunotherapeutic target. Its integration with established biomarkers could improve patient monitoring and treatment planning. At the same time, confounders such as parasitic exposure and microbiota composition must be carefully considered, as they may shape baseline antibody levels. Notably, prior studies on α-Gal–based cancer vaccines (18, 36) provide a strong rationale for exploring translational applications of this biomarker in HNC, emphasizing the value of expanding this line of investigation in well-controlled clinical settings.

## Data Availability

The original contributions presented in the study are included in the article/supplementary material. Further inquiries can be directed to the corresponding author.
